# “SLIME” on the probe cover provides better-quality ultrasound images without water-soluble gel

**DOI:** 10.1186/s40981-019-0263-9

**Published:** 2019-07-03

**Authors:** Yoshimune Osaka

**Affiliations:** Department of Anesthesiology, Kawasaki Municipal Hospital, 12-1 Shinkawa Street, Kawasaki-ku, Kawasaki City, Kanagawa 210-0013 Japan

To the Editor,

While performing ultrasonography for ultrasound-guided nerve block, an ultrasound transmission gel (water-soluble gel) is used to obtain good-quality ultrasound images. Air entering between the probe and probe cover interferes with the quality of the ultrasound images; however, it is sometimes difficult for beginners to prevent entry of air underneath the probe cover. On the other hand, the use of gel makes the probe sticky after the procedure. While a directly glued ultrasound probe cover without the use of gel has been reported [1], it is expensive.

We have found that the use of so-called “SLIME” (a mixture of polyvinyl alcohol and borax) is useful to obtain ultrasound images equivalent in quality to those obtained using gel.

We applied SLIME directly on a linear probe (S-Nerve HFL50x/15-6^®^, FUJIFILM Medical Co., Ltd., Tokyo, Japan) (Fig. 1a) and covered the probe and SLIME with a probe cover. Simple application of SLIME allows it to be placed firmly on the probe, and it is easy even for beginners to eliminate entry of air when it is covered with the probe cover (Fig. 1b). The quality of the ultrasound image using SLIME is equivalent to using gel (Fig. 1c, d). Preparation of SLIME is easy [2], as it is just a simple mixture of polyvinyl alcohol (included in laundry glue, KANEYO NOL^®^, KANEYO SOAP Co., Ltd., Tokyo, Japan) and borax (Housya®, KENEI Pharmaceutical Co., Ltd., Osaka, Japan), both of which are commercially available. The method of preparation of SLIME is as follows. First, mix equal amounts of laundry glue and water (50 ml each). Second, prepare the aqueous solution of borax with 2 g of borax in 25 ml of water. Third, pour this supernatant borax aqueous solution little by little into the water suspension of laundry glue and mix until the mixture becomes hard [2]. Although SLIME contains air, micro air might not interfere with the quality of ultrasound images. Subsequent to the ultrasound procedure, the SLIME can be peeled off easily from the probe without sticking which is different to gel (Fig. 1e, f) and is reusable. It is safe, unless the borax comes in direct contact with the skin.

**Fig. 1:**
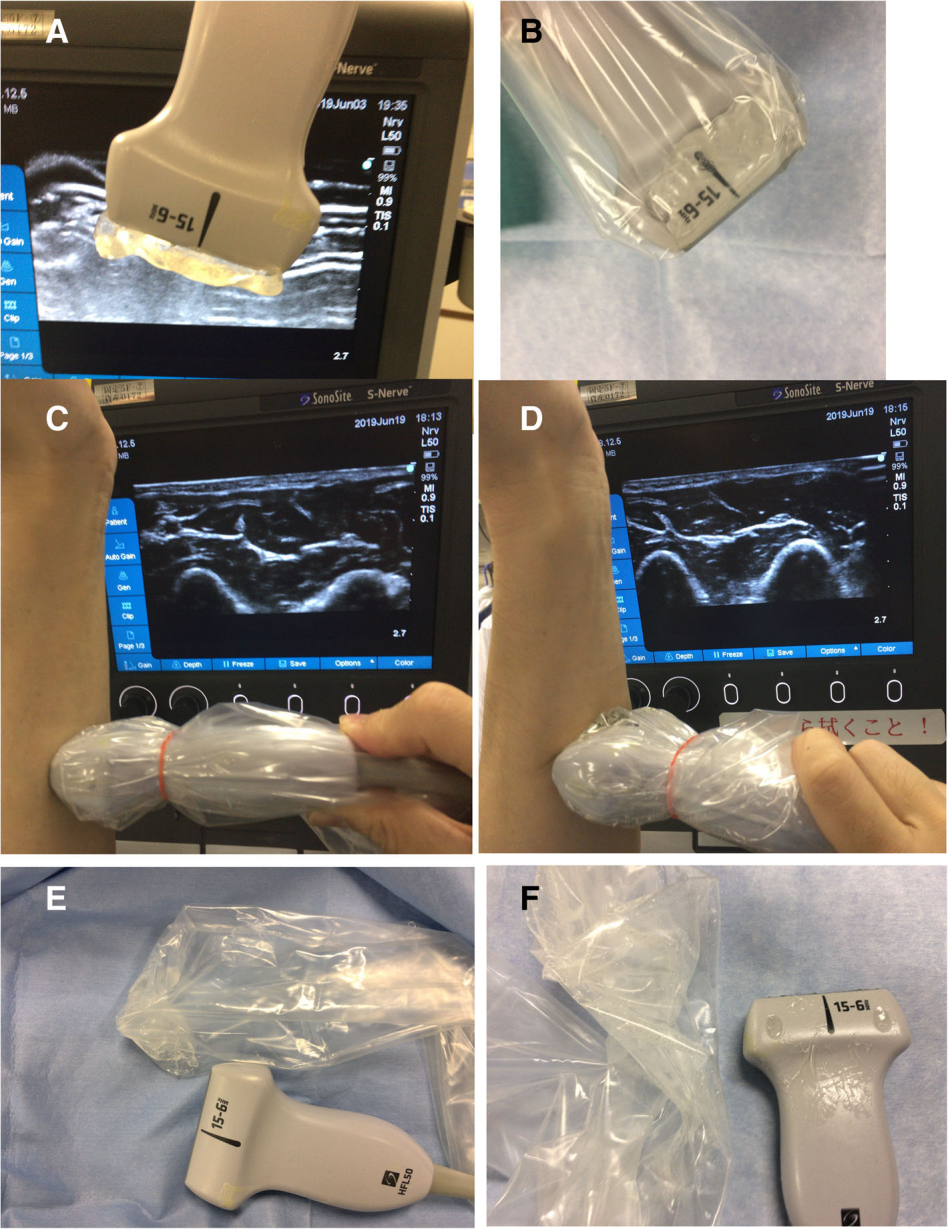
**a** Simple application of SLIME on the probe allows the SLIME to be placed firmly on the probe. **b** Application of SLIME on the probe allows entry of air between the probe and probe cover to be avoided. **c** The ultrasound image of the median nerve at the forearm obtained with the use of SLIME on the probe. **d** The ultrasound image with the use of water-soluble gel. **e** The probe remains smooth after removal of the probe cover using SLIME. **f** The probe after removal of the probe cover using gel

## Conclusions

The ultrasound image quality using SLIME is as equivalent as to that using water-soluble gel. In addition, SLIME as the probe cover might be easy even for beginners to eliminate entry of air and to treat the probe after the procedure.

## Data Availability

Not applicable.

## Abbreviations

SLIME: A mixture of polyvinyl alcohol and borax
